# Incidental perifissural nodules on routine chest computed tomography: lung cancer or not?

**DOI:** 10.1007/s00330-017-5055-x

**Published:** 2017-10-06

**Authors:** Onno M. Mets, Kaman Chung, Ernst Th. Scholten, Wouter B. Veldhuis, M. Prokop, Bram van Ginneken, Cornelia M. Schaefer-Prokop, Pim A. de Jong

**Affiliations:** 1 Department of Radiology, University Medical Center Utrecht, Heidelberglaan 100, 3584CX Utrecht, The Netherlands; 2 Diagnostic Image Analysis Group, Radboud University Nijmegen Medical Centre, Nijmegen, The Netherlands; 3 Department of Radiology, Radboud University Nijmegen Medical Centre, Nijmegen, The Netherlands; 4 Department of Radiology, Meander Medical Center, Amersfoort, The Netherlands

**Keywords:** Computed tomography, Adult, Solitary pulmonary nodule, Lung neoplasms, Guideline

## Abstract

**Objectives:**

Perifissural nodules (PFNs) are a common finding on chest CT, and are thought to represent non-malignant lesions. However, data outside a lung cancer-screening setting are currently lacking.

**Methods:**

In a nested case-control design, out of a total cohort of 16,850 patients ≥ 40 years of age who underwent routine chest CT (2004-2012), 186 eligible subjects with incident lung cancer and 511 controls without were investigated. All non-calcified nodules ≥ 4 mm were semi-automatically annotated. Lung cancer location and subject characteristics were recorded.

**Results:**

Cases (56 % male) had a median age of 64 years (IQR 59–70). Controls (60 % male) were slightly younger (*p*<0.01), median age of 61 years (IQR 51–70). A total of 262/1,278 (21 %) unique non-calcified nodules represented a PFN. None of these were traced to a lung malignancy over a median follow-up of around 4.5 years. PFNs were most often located in the lower lung zones (72 %, *p*<0.001). Median diameter was 4.6 mm (range: 4.0–8.1), volume 51 mm^3^ (range: 32–278). Some showed growth rates < 400 days.

**Conclusions:**

Our data show that incidental PFNs do not represent lung cancer in a routine care, heterogeneous population. This confirms prior screening-based results.

***Key Points*:**

• *One-fifth of non-calcified nodules represented a perifissural nodule in our non-screening population.*

• *PFNs fairly often show larger size, and can show interval growth.*

• *When morphologically resembling a PFN, nodules are nearly certainly not a malignancy.*

• *The assumed benign aetiology of PFNs seems valid outside the screening setting.*

## Introduction

Solid pulmonary nodules are a common finding in computed tomography (CT) of the chest, and management decisions regarding these nodules are encountered by many on an almost daily basis. The majority of solid nodules, however, will not represent a malignancy. Therefore, it is of utmost importance to reliably differentiate (potentially) malignant nodules from benign lesions. To guide this process, screening focused guidelines like Lung-RADS (Lung Screening Reporting and Data System) are available [1]. Additionally, for clinical use ACCP (American College of Chest Physicians) [2], Fleischner Society [3] and British Thoracic Society [4] guidelines have been issued.

A substantial subset of solid pulmonary nodules will represent perifissural nodules (PFNs), most likely representing intrapulmonary lymph nodes. Morphologically these are solid, homogeneous nodules with a smooth margin, and are oval or rounded, lentiform or triangular in shape [5–7]. It has been proposed to differentiate between typical and atypical PFNs on the one hand and non-PFN nodules on the other, based on the presence of fissural attachment and morphological characteristics [5]. Prior lung cancer-screening studies have shown that solid pulmonary nodules that conform to the definition of PFNs – either typical or atypical – do not represent or develop into lung malignancies over time, and should be regarded as non-suspect and benign lesions [5, 8].

Outside a screening setting, these perifissural nodules are also regularly found in daily routine imaging, and one may assume that they behave similarly. However, data on PFNs in routine chest imaging are currently lacking. Therefore, the purpose of our study was to assess the presence and behaviour of PFNs in a routine care, non-screening and heterogeneous population.

## Materials and methods

This retrospective study was reviewed by the institutional review board, who waived the need for informed consent.

### Subjects

This study is an ancillary project of a larger incident lung cancer study in non-screening subjects. For that study we retrospectively collected all subjects > 40 years of age who received a CT of the chest in our academic hospital between 2004 and 2012 (N=16,850). No selection was made based on imaging indication or in-/outpatient status. All eligible subjects were linked to the Dutch National Cancer Registry to identify those who did and did not develop lung cancer after the CT examination, until the end of 2014 (N=1,095 lung cancer cases). As we were interested in incident lung cancers, we excluded 867 subjects with a lung cancer diagnosis before or within 2 months after the CT (N=228 incident lung cancer cases). Applying a nested case-control design (ratio 1:3), we included these 228 subjects with a lung malignancy diagnosis (‘cases’), together with a randomly selected sample of 684 out of all subjects that did not develop a lung malignancy (‘controls’). We collected all available chest CT imaging up to 2 months before index date in cases, and the single oldest CT scan in controls. Lung cancer location and date of diagnosis were available from the National Cancer Registry. Patient characteristics and clinical information were obtained from the hospital radiology system.

### CT scanning

Scans were obtained at different scanners of the same vendor (Philips Healthcare, Best, The Netherlands). Since imaging was performed for various clinical indications, different scan protocols were applied. All scans were obtained with standard-dose protocols (90–140 kV at a median of 239 mAs). The vast majority of scans were reconstructed with thin-slice collimation (≤ 1 mm; 99 %), with a maximum slice thickness of 3 mm. Generally a sharp reconstruction filter was used (Philips-C; > 80 %).

### Nodule annotation

One of the authors, a radiology resident with a PhD in chest imaging and 7 years of experience in thoracic radiology, visually assessed all chest CT scans. Exclusion criteria were: insufficient image quality defined as mechanical ventilation, substantial consolidation or collapse, as well as severe motion/breathing artefacts that impeded reliable interpretation of lung tissue.

**Fig. 1:**
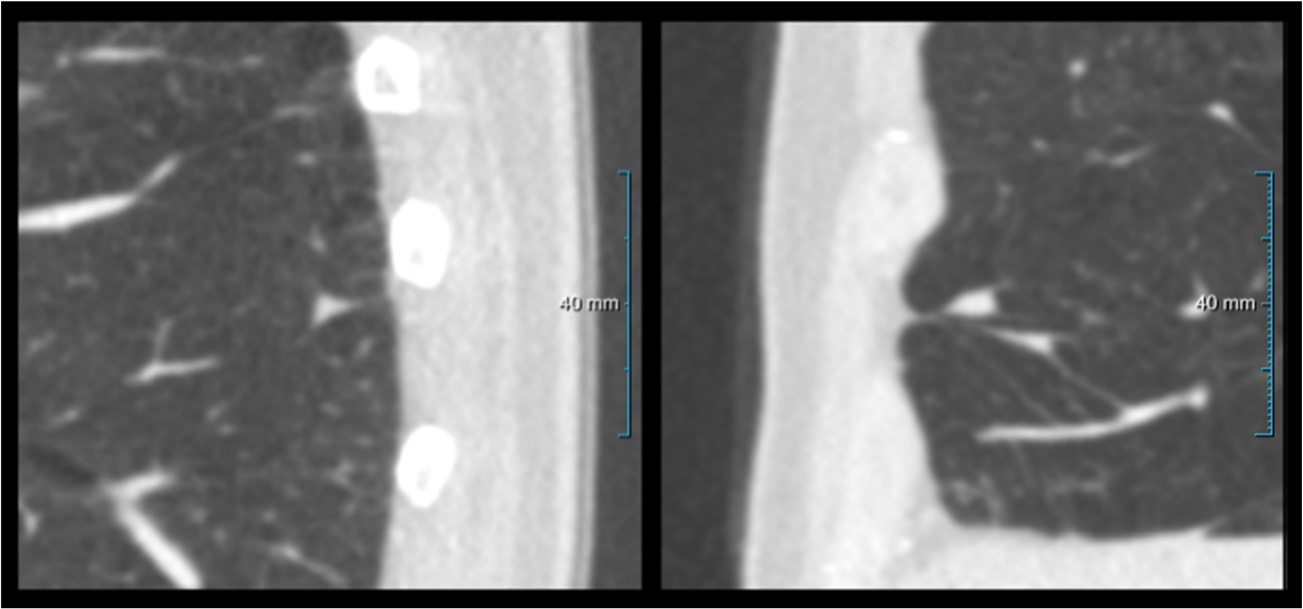
Example of two perifissural nodules (PFNs), morphologically presenting as a typical and an atypical PFN. **Left panel:** 70-year-old male, sagittal image. Triangular nodule in the left lower lobe attached to a fissure, meeting the definition of a typical PFN. **Right panel:** 69-year-old male, sagittal image. Fissure-attached nodule in the right middle lobe that is convex on one side and rounded on the other, meeting the definition of an atypical PFN

In the eligible CT scans all non-calcified nodules ≥ 4 mm were semi-automatically annotated by the observer using a dedicated nodule software workstation (CIRRUS Lung Screening, Diagnostic Image Analysis Group, Nijmegen, The Netherlands; Fraunhofer MEVIS, Bremen, Germany). Scans were read in a single session in which the software automatically provided suggestions for pulmonary nodules by placing a marker/box. The observer was able to accept, decline or adjust these. Additionally, the examination was thoroughly read for any additional nodules that were not picked up by the Cirrus software. The workstation enables image viewing in different window/level-settings, and provides reconstructions in all three orthogonal planes. Nodule type, volume, effective diameter, as well as location were scored for each annotated pulmonary nodule. In case of solid nodules a distinction was made between PFNs and non-PFN nodules, based on whether it morphologically showed a (peri)fissural or juxtapleural location (within 15 mm of the pleura), an oval or triangular shape, and smooth borders [5, 6]. Figure 1 shows two examples of a PFN, a group that by definition comprised both typical and atypical PFNs. Solid nodules that did not conform to the abovementioned definition of a PFN were regarded as non-PFN solid intrapulmonary nodules.

Besides the nodule characteristics, additional features were scored to evaluate potential interrelations. Emphysema was visually assessed. Adapted from Lynch et al. [9] – in which the authors describe the different types and extent of pulmonary emphysema – we assigned an overall score of emphysema extent on a four-point scale (none, mild, moderate and severe) [10]. Bronchial wall thickening was subjectively defined as thicker airway walls than normal, as there is no strict definition [10]. It was scored dichotomously as present or absent. Vascular calcifications in the coronaries and thoracic aorta were scored on a four-point scale (none, mild, moderate and severe), based on a previously described method [11]. Last, hilar or mediastinal lymphadenopathy was scored as present or absent, defined as nodes with a short-axis over 10 mm. To evaluate generalizability of visual scoring, a test sample of 34 scans (5 %) was also scored by two other authors (with over 10 and 20 years of experience, respectively).

### Statistical analysis

Straightforward descriptive statistics were used for this study. Age was compared between groups using Mann-Whitney U testing. Proportions were compared using chi-squared testing. Interobserver variability was calculated using Cohen’s kappa in dichotomous data, and using linear weighted kappa in ordinal data. Unless indicated otherwise, values given are medians with interquartile range (IQR). A *p*-value < 0.05 was considered statistically significant.

## Results

### Subjects

**Fig. 2:**
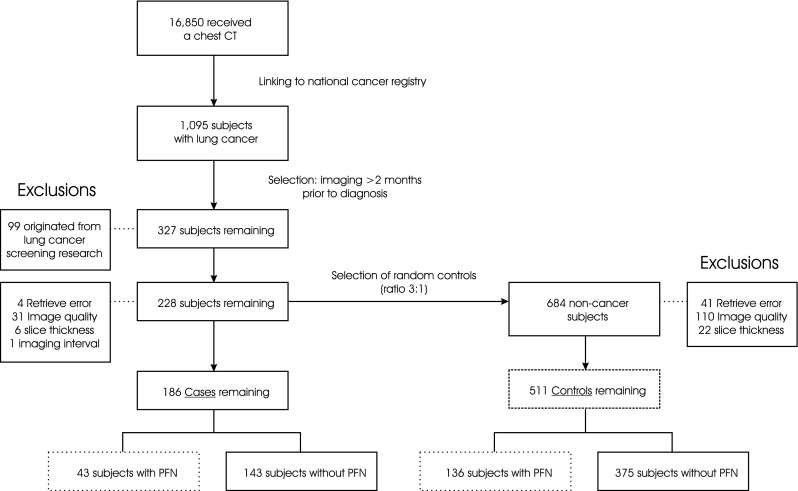
Flow chart of study population selection

After retrieval and visual assessment of the CT images, a total of 186 cases and 511 controls remained. This was due to exclusion of in total 42/228 cases and 173/684 controls, mainly due to insufficient image quality or slice-thickness. Figure 2 provides the flowchart of our study population selection. Cases were 56 % male, with a median age at the time of imaging of 64 years (IQR 59–70). Controls were 60 % male, and slightly younger at a median age of 61 years (IQR 51–70). Table 1 summarises basic study population characteristics.

**Table 1:** Study population characteristics

	Cases with incident lung cancers (N=186)	Controls without lung cancer during follow-up (N=511)	*p* value
Sex, N (%)			
Male Female	104 (56) 82 (44)	305 (60) 206 (40)	NS
Age, median (IQR)	64 (59–70)	61 (51–70)	<0.01
PFN, N (%)			
Present Absent	43 (23) 143 (77)	136 (27) 375 (73)	NS
Prior extrapulmonary malignancy, N (%)			
Present Absent	59 (32) 127 (68)	260 (51) 251 (49)	0.001

*IQR* interquartile range, *PFN* perifissural nodule.

### Pulmonary nodules

A total of 1,278 unique non-calcified nodules were found; 599 nodules in 178 cases (8/186 cases showed no nodules on available CT imaging) , and 679 nodules in 288 controls (223/511 controls showed no nodules on available CT imaging). In total 262 unique PFNs were annotated in 179 subjects (i.e. 69 unique PFNs in 43 cases, and 193 unique PFNs in 136 controls). Thus, one in every five nodules represented a PFN (21 %; 262/1278).

### Location, size and growth of perifissural nodules (PFNs)

**Fig. 3:**
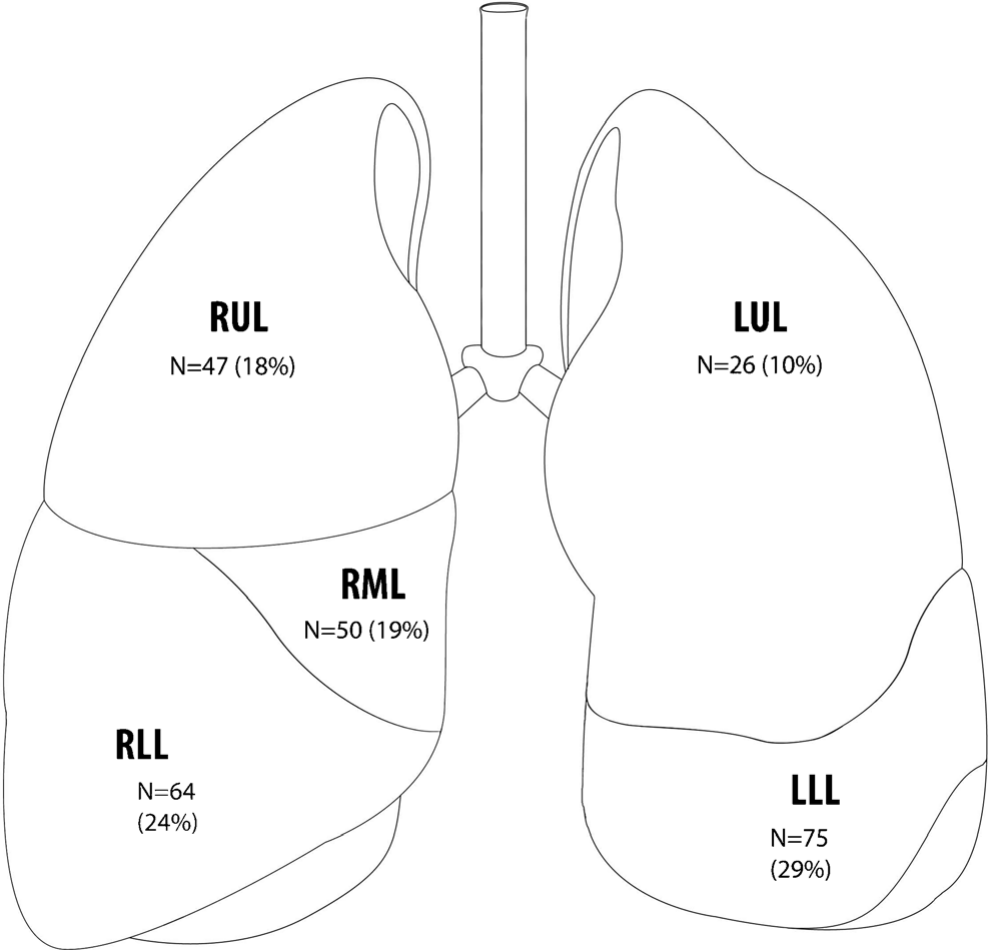
Location of perifissural nodules (PFNs) throughout the lungs

**Fig. 4:**
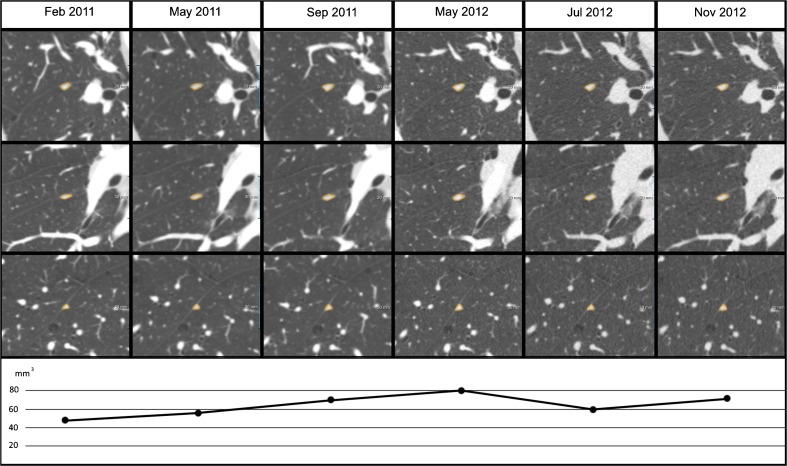
Example of growth in a perifissural nodule (PFN). A well-circumscribed, homogeneous, triangular-shaped solid nodule along the major fissure in the right lower lobe, presenting a PFN in an 58-year-old female without a prior malignancy. Nodule volume in the six consecutive CT scans ranged between 50 and 81 mm^3^ (diameter: 4.6–5.4) with a volume doubling time (VDT) of 361 days between CT2 and CT3, while a decrease with a VDT of -118 was seen between CT4 and CT5, as shown in the panels above

PFNs were most often located in the lower lobes, i.e. in 72 % (189/262), *p*<0.001. Figure 3 shows the distribution of PFNs throughout the lungs. The median diameter of all PFNs was 4.6 mm (range: 4.0–8.1, modus: 4.1), while volume median was 51 mm^3^ (range: 32–278, modus: 33). Volume doubling time (VDT) could be determined in a subset of 28 PFNs in 22 patients who were imaged multiple times during an interval of 424 days (IQR 187–585). Between the consecutive scans, two out of six growing PFNs showed a growth rate with a VDT of 400–600 days, while four showed a VDT of ≤ 400 days. Figure 4 shows an example of PFN growth. Three PFNs with more than one scan interval showed that none grew continuously, with volume decrease or stable volume in the other intervals. When using the Lung-RADS definition of growth (> 1.5 mm increase in diameter), only a single PFN fulfilled the criteria. This nodule decreased in size in another scan interval.

For those individuals with at least one PFN, the time interval between CT imaging and linkage with the cancer registry had a median of 53 months (IQR 30–85, range 6–126). None of the nodules annotated as PFN developed into or could be traced to a registered lung malignancy.

### Correlation with other chest CT parameters and patient characteristics

Interobserver agreement was moderate to substantial for the visual scoring (κ-values: 0.51–0.74). We found no significant difference in the presence or extent of emphysema, bronchial wall thickening, vascular calcifications or hilar/mediastinal lymphadenopathy between those with and without PFNs. PFN presence was also not different between those with or without any known extra-pulmonary malignancy, nor was there a relationship with age. However, there was a slight male preponderance (29 % vs. 22 %, *p*=0.04). When looking into the number of PFNs per subject it was found that those with hilar/mediastinal lymphadenopathy more often showed multiplicity (*p*<0.001 for > 2 PFNs).

## Discussion

Our study describes the presence and behavior of PFNs in a routine, non-screening population. Our results show that incidental PFNs on routine care chest imaging do not represent lung cancer, underlining prior results in lung cancer-screening participants.

PFNs are regularly encountered in daily practice, in a population that differs significantly from lung cancer-screening cohorts. It was previously reported that none of the PFNs that were evaluated in screening populations turned out to be malignant after several years of follow-up [5, 8]. It has since been assumed that this result could be extrapolated to clinical subjects, as shown by the incorporation into current nodule management guidelines for clinical use [3, 4]. However, to our knowledge this has never been tested. In the present study, out of the 262 evaluated PFNs, none were traced to a lung malignancy in follow-up. Although a single-centre study, our results support that PFNs are not lung cancer, a claim that is indeed valid in a broader spectrum of subjects. Our data therefore further strengthen the idea that when a lung nodule is encountered that morphologically conforms to a PFN, it is safe not to induce follow-up.

Currently, the ACCP guidelines [2] and the screening-focused Lung-RADS [1] do not separately discuss PFNs, and thus classify them as small solid pulmonary nodules at risk for lung cancer. Therefore, a PFN will induce unnecessary follow-up similar to a non-PFN nodule of the same size. The new Fleischner document and British Thoracic Society (BTS) guidelines do distinguish PFNs as a separate and non-malignant entity [3, 4]. However, the Fleischner document does not apply in subjects with a prior malignancy [3], patients which BTS does include, although based on low-level evidence. Depending on the practice, subjects with a prior malignancy can make up a substantial percentage of all subjects that receive CT imaging. Since our study also included subjects with prior malignancies, our data add significantly to the available literature by showing that in this subpopulation too PFNs do not represent lung cancer. It has to be emphasised, however, that our study is unable to indicate whether or not a PFN can contain a metastasis, since our outcome data only provided information on whether a patient developed a primary lung malignancy or not. Future research should elucidate this through prospective long-term follow-up of PFNs in patients with specific malignancies.

In the present study, we also looked into PFN size and growth. Most of the evaluated PFNs are rather small with a median of 4.6 mm and 51 mm^3^. It has to be noted that even this is an overestimation, given that we used a lower threshold of 4 mm in this study. Nevertheless, about a quarter of the PFNs were 80 mm^3^ or larger and about a third were 5 mm or larger, which are for example the cut-off values used for follow-up of solid nodules in the BTS guidelines [4]. Thus, it is not uncommon to encounter somewhat larger PFNs, and this should not be a reason to reject the diagnosis and suspect a lung cancer.

Although longitudinal imaging was available in only a small subset of cases, we found growth in some PFNs, sometimes even at a substantial growth rate. This is in line with previous screening-based literature [5, 8]. Growth is supposedly related to the fact that PFNs most likely represent intrapulmonary lymph nodes [3, 5], which may show reactive changes. While any growing lesion should raise concern and warrant closer inspection, our study underlines that PFN growth does not increase the likelihood of lung cancer, not even if the growth rate falls within the range generally accepted to indicate malignant growth [12]. Widespread knowledge on possible growth of PFNs might prevent unnecessary follow-up and additional imaging techniques, with associated costs and (radiation) burden. However, further evaluation of growth in clinically detected PFNs should be performed in future studies.

Another characteristic that we could reproduce in our non-screening study population is that PFNs are more often found in the lower part of the lungs [6, 8, 13]. This in contrast to an upper lobe location more often seen in lung malignancies [14]. Nevertheless, upper versus lower lobe location does not reliably differentiate between a benign or malignant nature of a nodule. This also applies for the (peri)fissural or juxtapleural location [3, 5, 15]. It is therefore important to emphasise that nodule morphology remains the only parameter for distinguishing benign PFNs from possibly malignant lesions.

Regarding nodule presence, we found that there was no relation to other chest CT biomarkers or age. In our cohort a slight male preponderence was found, a finding not reported in a previous screening-based study [8]. We cannot explain this finding with certainty, but believe that gender does not influence PFN characterisation. Given that PFNs are overall seen equally in cases and controls, in subjects with or without prior malignancies, and that there is no association with bronchitis, emphysema, vascular calcifications or mediastinal lymphadenopathy, it is likely a rather randomly occurring entity without prognostic or predictive value. We did found that subjects with hilar/mediastinal lymphadenopathy more often showed multiplicity of PFNs in the lung, which might represent their reactive nature.

The strength of this study is that it evaluates PFNs outside a lung cancer-screening setting, confirming in a daily-routine, heterogeneous population that they do not represent lung cancer. This includes patients with incident lung cancers as well as non-cancer subjects with or without prior extra-pulmonary malignancies.

Our study has several limitations. First, it had a retrospective study design with related heterogeneity of imaging protocols. For the sole purpose of PFN identification, however, we feel this is not a major factor. Nearly all included examinations were thin-slice images that allow good evaluation of small nodules in different reconstruction planes. Heterogeneity of imaging protocols may have had some influence on nodule segmentation. Given that PFNs were often small, limited segmentation differences may account for some of the observed growth. Second, results might have been influenced by the interpretation of lung nodule type in this single-observer study. We have given a clear definition of what was regarded a PFN versus a non-PFN lesion; however, some variation in interpretation is to be expected on a nodule-to-nodule basis. As far as we know, interobserver variability in PFN determination is currently not known, but it is well known from other lung nodule interpretation tasks that observer variation exists. Although some misclassification might thus be present in our study, separation between typical or atypical PFNs was not of importance in this study, and none of our PFNs turned out to be malignant. Third, our study had to rely on the National Cancer Registry for outcome data. This is less optimal than a study with several consecutive (screening) rounds and prospective follow-up. Due to this design, vital status was available for cases but not for controls, which overestimates their follow-up period. Nevertheless, the National Cancer Registry centrally registers all cancer cases in The Netherlands, irrespective of hospital. So, although control subjects may have died earlier than the end of our study period, we do know they did not develop lung cancer. Last, we do not have pathological evidence of the benign nature of all PFNs. Theoretically, a growing PFN could be based on a metastasis; however, evidence on that is anecdotal in the literature [16–18] and none grew continuously.

In conclusion, our study evaluates PFN presence and behaviour in a daily-routine, heterogeneous population. Results show that PFNs do not represent lung cancer, confirming prior results from lung cancer-screening studies. Given that small non-calcified nodules are a frequently encountered entity in every practice, and that a substantial percentage of these nodules represents a PFN, a leave-alone strategy substantially influences nodule management practice and reduces the number of follow-up CT examinations significantly.

## Abbreviations

CT: Computed tomography
PFN: Perifissural nodule
VDT: Volume doubling time
